# Protective Effects of *Lycium barbarum* Polysaccharides on Testis Spermatogenic Injury Induced by Bisphenol A in Mice

**DOI:** 10.1155/2013/690808

**Published:** 2013-12-26

**Authors:** Caili Zhang, Anzhong Wang, Xiaona Sun, Xiaocai Li, Xinghua Zhao, Shuang Li, Aituan Ma

**Affiliations:** College of Traditional Chinese Veterinary Medicine, Agricultural University of Hebei, Baoding 071001, China

## Abstract

To observe the effects of *Lycium barbarum* polysaccharides (LBP) on testis spermatogenic injuries induced by Bisphenol A (BPA) in mice. BPA was subcutaneously injected into mice at a dose of 20 mg/kg body weight (BW) for 7 consecutive days. LBP was administered simultaneously with BPA by gavage daily at the dose of 50, 100, and 200 mg/kg BW for 7 days. After treatment, the weight and the histopathology changes of testis and epididymis were examined; the contents of T, LH, GnRH, antioxidant enzyme, and malondialdehyde (MDA) in serum were detected; proapoptotic protein Bax and antiapoptotic protein Bcl-2 were also detected by immunohistochemical method. Results showed that the weights of testis and epididymis were all increased after supplement with different dosages of LBP compared with BPA group, and the activities of SOD and GSH-Px were significantly increased in LBP groups, while MDA contents were gradually decreased. Moreover, the levels of T, LH, and GnRH were significantly elevated in serum treated with 100 mg/kg LBP. LBP also shows significant positive effects on the expression of Bcl-2/Bax in BPA treated mice. It is concluded that LBP may be one of the potential ingredients protecting the adult male animals from BPA induced reproductive damage.

## 1. Introduction

There is a growing concern about the possible health threat posed by endocrine-disrupting chemicals (EDCs), which are substances involved in the environment, food, and consumer products that interfere with hormone biosynthesis, metabolism, or action resulting in a deviation from normal homeostatic control [1–3]. The well-documented issue of EDCs is related to xenoestrogens, antiestrogens, antiandrogens, disruption of thyroid function, and disruption of corticoid function, and other metabolic effects [4, 5]. EDCs can result in numerous adverse consequences in estrogen-targeted tissues, some of which may not be apparent until later in life. In addition to obesity and diabetes, reproductive damage has joined the list of adverse effects that have been associated with developmental exposure to environmental estrogens and other endocrine-disrupting chemicals [6, 7].

Bisphenol A (BPA) is an important monomer for producing plastics, like polycarbonates and epoxy resins; in addition, it is widely used in adhesives, flame retardants and dental composite fillings. Because of its wide spread applications, the potential hazard for human exposure has got a great awareness [8]. A study showed that the weights and coefficients of testis in BPA treated rats significantly decreased compared to the control. And BPA also improved the expression of Bax and decreased the expression of Bcl-2 [9]. The recent study has demonstrated that, after being treated with 100 mg/kg/day BPA from gestation day 0.5 to day 3.5 in C57BL6 mice, no embryo implantation was detected on gestation day 4.5 [10]. It has been reported that BPA may reduce testicular testosterone levels in mouse by adversely affecting both testis and pituitary systems which is similar to estradiol [11]. Our previous researches suggest that BPA would decrease the reproductive organ weights and coefficients, downregulate the levels of T and LH, and damage the spermatogenic capability in adult male mice [12]. The harmfulness of BPA as well as many other EDCs on reproductive system has been tested by a number of studies, while the data for how to reverse the damage is very limited.

*Fructus lycii* has been used in China over thousand years as a herbal medicine to promote fertility and has been included in most fertility promoting Chinese herbal remedies. *Lycium barbarum* polysaccharide (LBP) is the most important functional ingredient in *Fructus lycii*. Many studies have shown that LBP may regulate immune system and play an important part in antitumor, antioxidation, antiradiation, antihypertension, antihyperlipemia, and other pharmacological properties [13]. Recently, Luo and his colleagues reported that LBP provided a protective effect against the testicular damage induced by heat exposure and also had a dose-dependent protective effect against DNA oxidative damage in mouse testicular cells induced by H_2_O_2_ [14].

In recent years, researchers mainly focus on the protective effect of LBP on oxidative stress of animals. However, there have been few reports on whether LBP draws some beneficial effects on BPA induced injuries in testis. The present study was designed to investigate whether LBP has a protective effect against BPA induced negative changes in histological structure of testis, which may be associated with oxidative stress, decreased hormone level, and the expression of apoptosis proteins in adult male mice.

## 2. Materials and Methods

### 2.1. Chemicals and Reagents

BPA and olive oil were purchased from Sigma Co. (USA). *Lycium barbarum* polysaccharides (LBP⩾78.5%) were purchased from Qufu Natural Green Engineering Co. (China). Anti-Bax polyclone antibody and anti-Bcl-2 polyclone antibody were purchased from Beijing Biosynthesis Biotechnology Co. LTD (China). DAB kit was obtained from Beijing Zhongshan Golden Bridge Biotechnology Company (China). Superoxide dismutase (SOD), glutathione-peroxidase (GSH-Px), and malondialdehyde (MDA) assay kits were obtained from Jiancheng Bioengineering Institute (Nanjing, China). Testosterone (T), luteinizing hormone (LH), and gonadotropin-releasing hormone (GnRH) assay kits were purchased from RB Co. (USA).

### 2.2. Animals and Treatment

Fifty adult male mice with average weight of 25 ± 0.45 g were purchased from the Experimental Animal Center of Hebei Medical University (China). All animals had access *ad libitum* to rodent feed and water in glass bottles with rubber stoppers. Mice were kept in a room that maintained a temperature range of 21-22°C and with a light-dark cycle of 12 : 12 hours. After an adaptive period of 1 week, they were randomly divided into 5 groups (10 mice/group), namely, the control group (A), the BPA group (B), the low-dose (50 mg/kg) LBP group (C), the medium-dose (100 mg/kg) LBP group (D), and the high-dose (200 mg/kg) LBP group (E). Except for the mice in control group (A), which were administrated with olive oil (the solvent of BPA), mice in other 4 groups were administrated with BPA at 20 mg/kg BW [12]. Meanwhile, the mice in groups C, D, and E were administrated with 50, 100, and 200 mg/kg BW of LBP daily, respectively, for 7 days, and the mice in groups A and B were given equivalent amount of normal saline. Animal handling and treatment were performed in compliance with Chinese national guidelines. These tests were made with 3 replications.

### 2.3. Body Weight and Coefficient

After 1-week treatment, the mice were sacrificed under deep 2% Nembutal anesthesia (40 mg/kg BW) and the body weight, the weights of testes and epididymises were recorded. The organ coefficients were calculated according to organ weight/body weight ×100%.

### 2.4. Histological Evaluation

Testes were fixed in Bouin's solution and embedded in paraffin. Histological sections (5 *μ*m) were cut and mounted on glass slides, then deparaffinized, and rehydrated in a graded series of ethanol, followed by staining with hematoxylin and eosin, and slides were then dehydrated, cleared, and mounted for micromorphological evaluation [15].

### 2.5. Assay of SOD, GSH-Px, and MDA

After blood samples were collected (ten samples in every group), centrifugating them at 3000 rmp × 10 min⁡ to get serum for test, SOD was measured by xanthine oxidase method which was based on the ability to inhibit oxidation of oxyamine by the oxyamine-xanthine oxidase system; GSH-Px was measured by DNTB colorimetric method through the consumption of glutathione; MDA was measured by thiobarbituric acid (TBA) reaction. Values were calculated using optical density (550 nm for SOD, 412 nm for GSH-Px, and 532 nm for MDA) and expressed as units (U) per mg protein for SOD, GSH-Px, and nmol/mg protein for MDA.

### 2.6. The Serum Levels of T, LH, and GnRH

T, LH, and GnRH were assayed in the serum (ten samples in every group), all standards and samples were added in duplicate to the microelisa stripplate. Firstly, 50 *μ*L standard was added to standard well, while 10 *μ*L testing sample and 40 *μ*L sample diluent were added to testing sample well, and nothing was added to the blank well, then adding 100 *μ*L HRP-conjugate reagent to each well, covering the microelisa stripplate with an adhesive strip and incubating it at 37°C for 60 min, then aspirating each well, and washing the well with washing solution completely (removing the liquid at each step is essential). Furthermore, adding 50 *μ*L chromogen solution A and 50 *μ*L chromogen solution B to each well and then gently mixed and incubated it at 37°C for 15 min (protect from light). At last, 50 *μ*L stop solution was added to each well, and the color in the wells changed from blue to yellow, reading the optical density (O.D) at 450 nm using a microtiter plate reader.

### 2.7. Expression of Bax and Bcl-2 by Immunohistochemistry

Immunohistochemical staining was conducted on 5 *μ*m sections of the tissue microarray blocks. The paraffin sections were mounted on glass slides, deparaffinized, and rehydrated in a graded series of ethanol, followed by microwave antigen retrieval. Endogenous peroxidase activity was blocked using 3% hydrogen peroxide. The sections were incubated overnight at 4°C using primary antibodies (rabbit anti-mouse Bax and Bcl-2 polyclone antibody). The second antibody is biotinylated goat anti-rabbit IgG, and immunostaining was conducted using the DAB kit. The sections were then counterstained with hematoxylin and were then dehydrated, cleared, and mounted. The primary antibody was replaced by PBS as negative control. Selecting 10 circular seminiferous tubules on each slice, each treatment group was selected 100 circular seminiferous tubules at 400 magnification to count the number of positive cells.

### 2.8. Statistical Analysis

The data were analyzed by one-way analysis of variance, using the SPSS 16.0 software. All data were presented as means ± SD. *P* < 0.05 and *P* < 0.01 and were considered statistically significant.

## 3. Results

### 3.1. Weights and Coefficients of Testis and Epididymis

Compared to the control group, the weights and coefficients of testis and epididymis in the BPA group were overly atrophic (13.0% and 34.0% of weight, 19.1% and 42.8% of coefficient, *P* < 0.01). After LBP treated, the weights and coefficients of testis almost recovered to normal level. The weights and coefficients of epididymis in LBP treated groups were also ameliorated compared to the BPA group, especially in the group of 100 mg/kg LBP. However, the weights and coefficients of epididymis in all LBP treated groups (C, D, and E) were unable to return to the normal (Table 1).

### 3.2. Histopathological Observation of Testis

**Figure 1:**
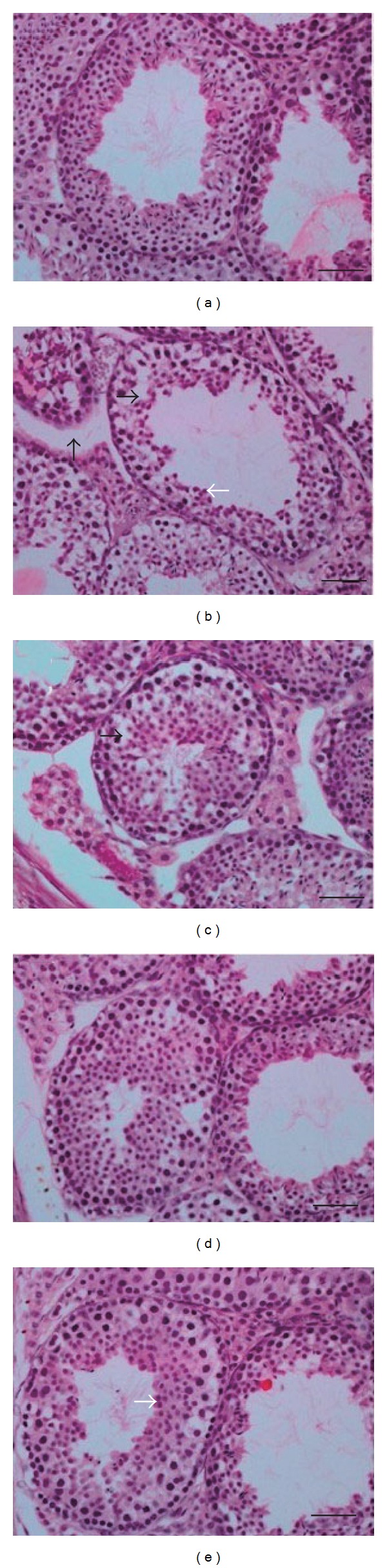
Development of spermatogenic cells in seminiferous tubules of mice testis (Bar = 100 *μ*m). (a) Control group: the histological structure of seminiferous tubules is normal with 5–7 layers of closely and orderly arrayed spermatogenic cells. (b) BPA group: some of the spermatogenic cells desquamate or vanish. The spermatogenic cells array loosely and disorderly. There are gaps between spermospores and primary spermatocytes. (c) 50 mg/kg LBP (L) group: the spermatogenic cells are less closely arrayed, and some of the spermatogenic cells desquamate. (d) 100 mg/kg LBP (M) group: the spermatogenic cells are less closely arrayed and different stages of spermatogenic cells can be identified. (e) 200 mg/kg LBP (H) group: the spermatogenic cells in this group are more closely and tightly arrayed than cells in 50 mg/kg and 100 mg/kg group. The histological structure of seminiferous tubules is clear. The gaps between cells were larger than usual (the thick black arrow); the seminiferous tubules spaces were bigger (the thin black arrow); the spermatogenic cells array loosely and disorderly, and some spermatoggenic cells desquamated (the thick white arrow); the cells array more orderly and closely than other groups except the control group (the thin white arrow).

In the control group, the histological structure of seminiferous tubules was normal and the spermatogenic cells were tightly and organized. Different stages of spermatogenic cells could be identified clearly with small and round spermatogonia closing to the basement membrane of seminiferous tubules and the primary spermatocyte, spermatid, and sperms distributed orderly toward the lumen (Figure 1(a)). In BPA group, most of the seminiferous tubules contained less spermatogenic cells, only 2-3 layers of cells, or desquamated cells. No spermatogenic cells were observed in some tubules. Large vacuolization could be seen (Figure 1(b)). In 50 mg/kg LBP group, the spermatogenic cells were observed less closely (Figure 1(c)). The spermatogenic cells were less closely arrayed but different stages of spermatogenic cells could be identified in 100 mg/kg LBP group (Figure 1(d)). Compared with 50 mg/kg group, the histological structure of seminiferous tubules in 200 mg/kg LBP group was clearer and spermatogenic cells were more closely and orderly arranged. Differentiation stages of spermatogenic cells could be identified clearly in 200 mg/kg LBP group (Figure 1(e)).

### 3.3. Effect of LBP on SOD, GSH-Px, and MDA in BPA Treated Mice

The activities of SOD and GSH-Px were significantly (*P* < 0.01) decreased from 138.24 U·mg prot^−1^ and 48.08 U·mg prot^−1^ in control group to 85.61 U·mg prot^−1^ and 31.59 U·mg prot^−1^ in BPA group. On the contrary, MDA content was elevated significantly (*P* < 0.01) from control group 0.73 nmol·mg prot^−1^ to 2.31 nmol·mg prot^−1^in BPA group. Compared to BPA group, the activities of SOD and GSH-Px in all LBP-treated groups were significantly elevated (*P* < 0.01). MDA contents in 100 and 200 mg/kg LBP treated mice were decreased significantly.

### 3.4. Effect of LBP on the Serum Hormone Levels in BPA Treated Mice

**Figure 2:**
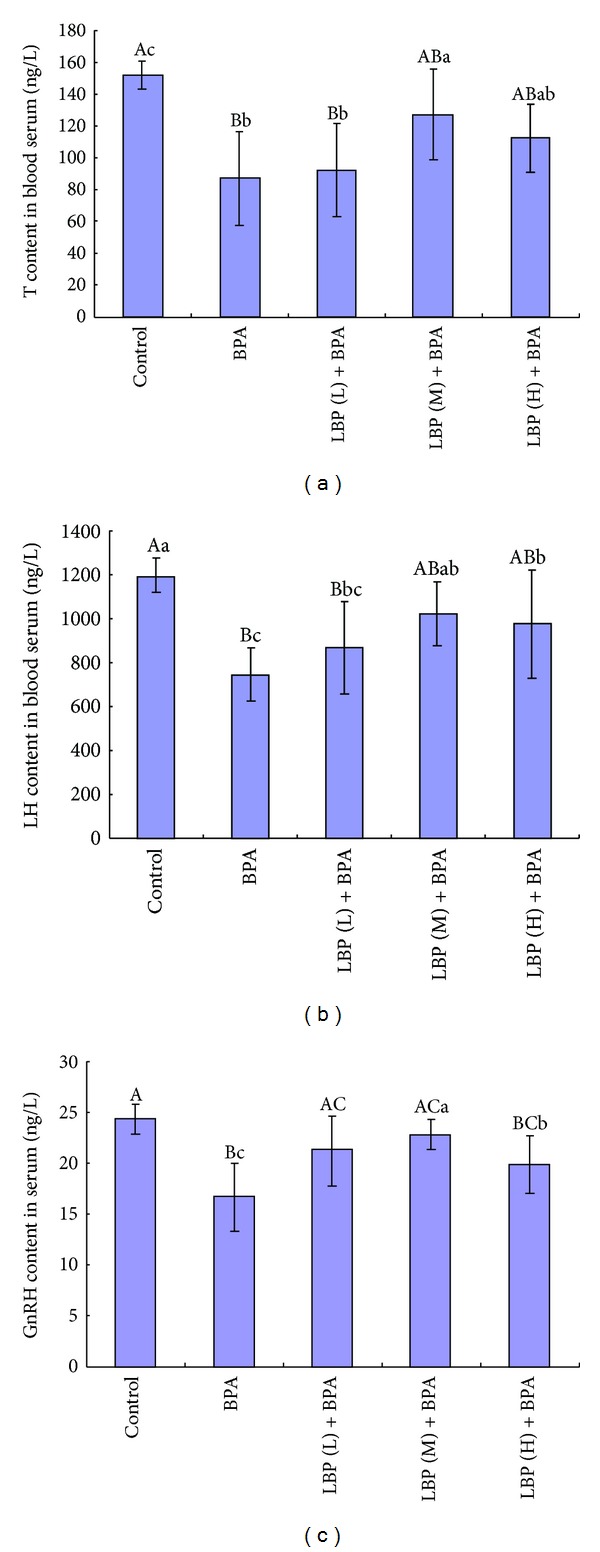
Effects of LBP on serum levels of T, LH, and GnRH in mice. (a) The content of T in different groups. (b) The content of LH in different groups. (c) The content of GnRH in different groups.

Compared to the control group, the serum contents of T, LH, and GnRH were significantly decreased (*P* < 0.01) in BPA treated group (Figure 2), while after adding LBP, especially in 100 mg/kg LBP group, serum levels of T, LH, and GnRH increased more significantly than that in BPA group.

### 3.5. Expression of Bax and Bcl-2 in Spermatogenic Cells

**Figure 3:**
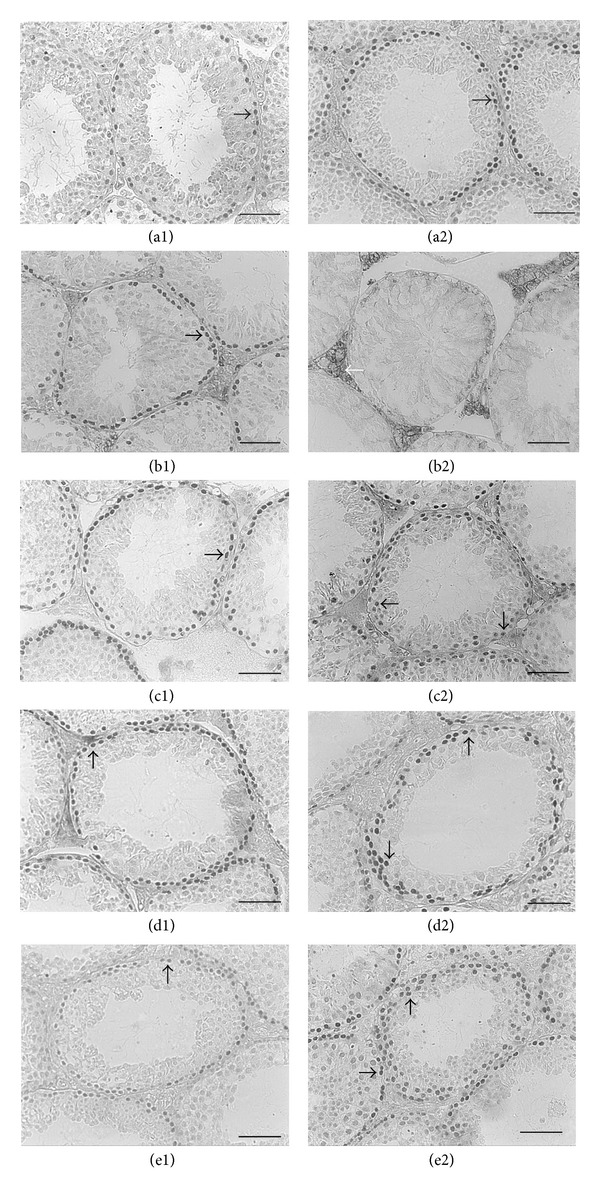
Expression of Bcl-2 and Bax proteins in spermatogenic cells in mice (Bar = 100 *μ*m). (a1)–(e1) Expression of Bax protein. (a2)–(e2) Expression of Bcl-2 protein. (a) Control; (b) BPA group; (c) 50 mg·kg^−1^ LBP group; (d) 100 mg·kg^−1^ LBP group; (e) 200 mg·kg^−1^ LBP group. The thick black arrow indicates spermatogenous cell; the thin black arrow indicates the primary spermatocyte; the white arrow indicates Leydig cell.

In the control group, the positive expression of Bcl-2 mainly located in spermatogenous cells, while in the BPA group it was mainly expressed in Leydig's cells and Sertoli cells (Figures 3(a1)–3(e1)). The expression of Bcl-2 was statistically decreased in BPA treated group compared with that in the control group. After LBP treatment, the positive expression highly located in spermatogenous cells and primary spermatocytes, and the expression of Bcl-2 increased in a dose-dependent manner of LBP, especially in the 100 mg/kg and 200 mg/kg group compared to the BPA group (Table 3).

The proapoptotic protein Bax was mainly expressed in cytoplasm of spermatogenous cells in all groups (Figures 3(a2)–3(e2)) but highly expressed in the BPA group, which is significantly higher (24.4%) than that in the control group (*P* < 0.01). After adding LBP, the expression of Bax decreased with the increasing dose of LBP, especially in the 200 mg/kg LBP group compared to the BPA group (Table 3).

As for the ratio of Bcl-2/Bax, it is significantly decreased to 0.9150 in the BPA group versus the control value of 1.3841. However, the ratio in LBP groups showed some increasing tendency with dose-dependent manner (Table 3).

## 4. Discussion

Endocrine disruptors are substances commonly encountered in every setting and condition in the modern world. It is virtually impossible to avoid the contact with these chemical compounds in our daily life. Molecules defined as endocrine disruptors constitute an extremely heterogeneous group and include synthetic chemicals used as industrial solvents/lubricants and their byproducts. Natural chemicals found in human and animal food (phytoestrogens) also act as endocrine disruptors [16]. It may damage the development of the reproductive system and associated organs [17, 18]. There is some controversy as to the effects and mechanisms by which EDCs acted [19, 20], and the most accepted hypothesis holds that EDCs interfere with steroid hormone action through disruption of steroid biosynthesis, the hormone balance, and signaling pathways of downstream consequences.

In present experiment, after being treated with BPA at 20 mg/kg body weight (BW) for 7 days in adult male mice, the weights and coefficiencies of testis and epididymis in BPA group were significantly decreased (*P* < 0.05), and the histological structure of seminiferous tubules was atrophic severely compared to the control group, the normal array of tubules was disrupted, the spermatogenic cells were reduced, the cells arrayed loosely and disorderly, and the space between seminiferous tubules was larger. It indicated that BPA may damage both testis and epididymis and cause the histological structure changes of testis and epididymis (Table 1), through decreasing the number of sertoli cells, and/or disrupting the function of hypothalamic-pituitary-gonadal axis [7]. A number of studies have shown BPA can decrease the weights and coefficients of testis and epididymis, and the quality of Leyding and spermatogenic cells, it may induce tumors of testis and cryptorchidism as well [21, 22].

In recent decades more attention has been paid to the medicinal plants which possess high efficiency and low toxicity. *Fructus lycii* has been well-known in traditional Chinese herbal medicine for centuries and nowadays has been widely used as a popular functional food, with a large variety of beneficial effects. It has been known for decades that LBP is a biologically active component of *Fructus lycii* with potential pharmacological and biological functions including antioxidant and anti-infertility activities [13, 14]. Taking *Fructus lycii* and LBP orally is the best way to exert their beneficial effects both for human and animal without concerning about the endotoxin [23, 24]. In present study, after supplement with LBP in BPA treated mice, the weights and coefficients of testis and epididymis recovered toward the control, especially for the testis (Table 1). The histological structure of seminiferous tubules was also ameliorated. In 200 mg/kg LBP group, the histological structure of seminiferous tubules was clear and the spermatogenic cells were more closely and tightly arrayed than cells in 50 mg/kg LBP and 100 mg/kg LBP group (Figure 1). It revealed that LBP can protect the testis and epididymis from BPA induced injuries, the mechanism of LBP may, firstly, through gonadotropin-like effect promote the hypophysis secretes gonadal hormone and regulate the hypothalamic-pituitary-gonadal axis in a multiple manner. Secondly, LBP may reduce the damage of BPA on spermatogenic cells through suppressing the damage of lipid peroxidation and other peroxide radicals on DNA. Other study also has shown that LBP can increase the weights and coefficienies of testis to normal level [25].

Recently, much attention has been focused on oxidative stress as a major factor of male infertility which is mediated by reactive oxygen species (ROS) and lipid peroxidation [26, 27]. It has been proposed that oxidative stress is one of the fundamental molecular mechanisms involved in xenobiotic-induced toxicity [28]. Many ways and mechanisms are involved inside the body to protect cells against damage caused by oxidative free radicals, including SOD and GSH-Px. MDA is the main product of lipid peroxidation which plays a big role in cytotoxicity [29], and the content of MDA also increased in high fat diet mice [30]. After being treated with BPA, the serum levels of SOD and GSH-Px were significantly decreased (*P* < 0.01), while the MDA level was increased significantly (*P* < 0.01) versus the control group (Table 2). These changes suggested that severe oxidative damages were induced by BPA. Kabuto's [31] study indicated that during embryonic/fetal exposure to BPA, the level of tissue SOD and GSH decreased, it indicated that damage induced by BPA may be related to ROS and lipid peroxidation. However, in LBP group, the serum levels of SOD and GSH-Px were increased (*P* < 0.05), while MDA was decreased (*P* < 0.05) (Table 2) which corresponds to the study made by the group of Wang [32], it showed that LBP3 would downregulate the activity of lipid peroxidation via upregulating the content of SOD and GSH-Px, the active of LBP on serum SOD, GSH-Px and MDA of BPA damaged mice may be related to it. LBP has been used as an antioxidant for a long time, and it can improve the antioxidation function of cells and eliminate the oxygen radical and decrease the oxidative damage [33]. One study has shown that LBP can improve the levels of GSH-Px and SOD in normal mouse, and decrease the level of MDA in liver of CCL_4_ treated mice [34].

Earlier observations similarly suggested that the neonatal exposure of rats to BPA can lead to significant imbalances of the hormonal in the experimental animals, which indicates that BPA has the potential to disrupt the hypothalamus-pituitary-testicular axis. The alteration in endocrine regulation and impairment of the hypothalamus-pituitary-testicular axis were also evident at the testicular level, wherein the presence of sloughed spermatozoa was seen in the seminiferous tubules [35]. In the present study, compared to control group, the serum contents of T, LH, and GnRH in BPA group decreased significantly (*P* < 0.01), while after being treated with LBP, the levels of T, LH, and GnRH increased especially in 100 mg/kg LBP group. It indicated that LBP may protect mice from BPA induced damage (Figure 2). Another study also showed that LBP can increase the levels of T, LH, and FSH in mouse serum treated by warm bath [36].

Bcl-2 family proteins consist of both proapoptotic and antipoptotic proteins. Antiapoptotic Bcl-2 family proteins, such as Bcl-2, prevent the release of apoptogenic molecules from the intermembrane space of mitochondria, whereas proapoptotic members of the Bcl-2 family, such as Bax, induce those events [37]. Mitochondria may be one of the most important locations for apoptosis [38], which has close relationship with the levels of Bax and Bcl-2 [39]. This study has shown that the expression of Bcl-2 was diminished in BPA group (*P* < 0.01), while the expression of Bax was significantly increased (*P* < 0.01) (Table 3). It suggested that BPA can diminish the expression of Bcl-2, increase the expression of Bax, and thus decrease the ratio of Bcl-2/Bax. Presnet study showed that the expression of Bcl-2 was upregulated with the dose increasing of LBP, especially in the 100 mg/kg and 200 mg/kg LBP groups compared to the BPA group (*P* < 0.05). After LBP treatment, the expression of Bax was downregulated with the dose of LBP increasing, especially in the 200 mg/kg LBP group compared to the BPA group (*P* < 0.01), and the ratio of Bcl-2/Bax in LBP groups showed a tendency of dose-dependent manner (Table 3). A number of studies have shown that LBP can increase the expression of Bcl-2 and decrease the expression of Bax and increase the ratio of Bcl-2/Bax [40].

## 5. Conclusions

In summary, present experiment has demonstrated that BPA may disturb the reproductive system function of adult male mice. While LBP, a long been used Chinese traditional medicine, can mitigate the BPA injuries through suppressing the oxidative stress and regulating the ratio of Bcl-2/Bax in adult male mice. As for the dosage of LBP, supplement with 100 mg/kg of LBP has the best positive effects among the three dosages on the organ coefficients of testis and epididymis and the oxidation resistance, as well as the level of T, LH and GnRH, while supplement with 200 mg/kg of LBP has the better result on the histological structure of testis and on the ratio of Bcl-2/Bax. To our knowledge, the present study is the first report that shows the protective effect of LBP against testicular damage induced by environmental estrogens.

## Figures and Tables

**Table 1:** Effect of LBP on testicular weight, epididymal weight, and organ coefficients in mice.

Groups (mg/kg) (*n* = 10)	Testicular weight (g)	Testis coefficients (%)	Epididymal weight (g)	Epididymis coefficients (%)
A (control)	0.23 ± 0.03^a^	0.68 ± 0.08^a^	0.103 ± 0.020^Aa^	0.325 ± 0.066^A^
B (BPA 20)	0.20 ± 0.02^b^	0.55 ± 0.07^b^	0.068 ± 0.09^Bc^	0.186 ± 0.024^Ba^
C (BPA 20 + LBP 50)	0.22 ± 0.03^a^	0.61 ± 0.11^ab^	0.076 ± 0.013^B^	0.208 ± 0.045^B^
D (BPA 20 + LBP 100)	0.22 ± 0.03^a^	0.66 ± 0.07^ab^	0.086 ± 0.0126^ABb^	0.240 ± 0.036^Bb^
E (BPA 20 + LBP 200)	0.22 ± 0.02^a^	0.67 ± 0.15^a^	0.082 ± 0.003^B^	0.214 ± 0.038^B^

The different lowercase and capital letters that followed the data in the same column showed significantly difference at 0.05 and 0.01 levels, respectively.

**Table 2:** Effect of LBP on the contents of SOD, GSH-Px, and MDA in the testis of mice.

Groups (mg/kg) (*n* = 10)	SOD activities /U·mg prot^−1^	GSH-Px activities /U·mg prot^−1^	MDA contents /nmol·mg prot^−1^
A (control)	138.24 ± 6.42^A^	48.08 ± 5.33^Aa^	0.73 ± 0.05^Bc^
B (BPA 20)	85.61 ± 8.32^B^	31.59 ± 2.49^B^	2.31 ± 0.26^Aa^
C (BPA 20 + LBP 50)	124.90 ± 5.79^ACa^	38.43 ± 1.72^Cab^	1.49 ± 0.25^ACab^
D (BPA 20 + LBP 100)	133.28 ± 12.14^ACb^	44.08 ± 2.43^ACab^	1.53 ± 0.18^ACb^
E (BPA 20 + LBP 200)	129.45 ± 13.66^A^	41.37 ± 1.23^ACb^	1.23 ± 0.06^BCab^

The different lowercase and capital letters that followed the data in the same column showed significant difference at 0.05 and 0.01 levels, respectively.

**Table 3:** Expression of Bcl-2, Bax, and the ratio of Bcl-2/Bax in different groups.

Groups (mg/kg)	Bcl-2	Bax	Bcl-2/Bax
A (control)	64.5278 + 2.3649^ABa^	47.2299 + 1.0989^Aa^	1.3841
B (BPA 200)	56.6913 + 1.8175^Ab^	62.5213 + 1.4236^Bb^	0.9150
C (BPA 20 + LBP 50)	57.2136 + 1.3980^Ab^	56.6381 + 3.5688^ABb^	0.9735
D (BPA 20 + LBP 100)	64.2847 + 2.6425^ABa^	55.9458 + 2.8589^ABb^	1.1381
E (BPA 20 + LBP 200)	67.2050 + 2.5054^B^	53.0039 + 1.8613^Aab^	1.2706

The different lowercase and capital letters that followed the data in the same column showed significant difference at 0.05 and 0.01 levels, respectively.
